# Investigating the effect a single dose of cannabidiol has on measures of stress in cats when being transported in a carrier and meeting a novel person in an unfamiliar environment

**DOI:** 10.3389/fvets.2024.1476296

**Published:** 2024-11-04

**Authors:** Jennifer E. Weller, Hannah E. Flint, Alysia B. G. Hunt, Zack Ellerby, Tammie King

**Affiliations:** Waltham Petcare Science Institute, Waltham on the Wolds, United Kingdom

**Keywords:** Cannabidiol, cbd, cat, stress, anxiety, behaviour, animal welfare

## Abstract

Domestic cats (*Felis Catus*) are often exposed to stimuli that have the potential to negatively impact their welfare. These can include situations such as veterinary visits, travel, changes to their home environment, and interactions with unfamiliar people or pets. Cannabidiol (CBD)-infused pet products have grown in popularity in recent years, as pet owners search for ways to calm and relax their pets. However, research into the pharmacokinetic properties of CBD in cats is limited and investigations into its efficacy are in their infancy. The present study aimed to explore the effect of a single 4 mg/kg bodyweight dose of a THC-free CBD distillate on measures of stress in cats when experiencing a composite stress-paradigm, consisting of cat carrier travel and exposure to a novel person within an unfamiliar environment. Physiological and behavioural indicators of stress were collected pre-, during, and post-testing. No significant effect of CBD was observed on serum cortisol, IgA, or glucose, either before or immediately after the stress-paradigm (all *p* > 0.05). This was true despite cortisol being shown to significantly increase post-test for both treatments (both *p* < 0.001), suggesting that travel and meeting a novel person successfully induced a stress response within this population of cats. No significant differences in any measures of cat behaviour, including latency to approach the novel person, were observed between treatments (all *p* > 0.05). Overall, no influence of CBD was observed in cats, suggesting further research into appropriate dosage, delivery matrices, and other conditional factors, such as individual coping styles, should be considered.

## Introduction

Cannabidiol, commonly referred to as CBD, is the second most abundant phytocannabinoid found within the multi-purpose cannabis (*Cannabis sativa*) plant (1). When present within the body, CBD activates the endocannabinoid system, a system observed to be particularly pervasive in mammalian species (2). While the full mechanisms of CBD pharmacology have not yet been determined (1, 3), many studies have indicated that CBD is effective at preventing and managing multiple neurodegenerative disorders in humans (reviewed by Iuvone et al. (3)), as well as other physical and mental health conditions (4, 5). Unlike Δ^9^-tetrahdrocannabinol (THC), CBD is widely recognised to be a non- or minimally psychoactive molecule (3, 6), making it potentially suitable for use as a medical intervention. For example, a review by Blessing et al. (4) reported preclinical evidence of CBD as a treatment for generalised anxiety disorder, panic disorder, social anxiety disorder, obsessive-compulsive disorder, and post-traumatic stress disorders. Furthermore, patients experiencing multiple sclerosis-related central neuropathic pain reported reduced pain and fewer sleep disturbances when treated with a CBD/THC combination (5). CBD may also modulate the neurobiological process involved in Pavlovian fear conditioning and contextual fear memory processing (7), with evidence suggesting that the administration of CBD mitigates the increase in freezing behaviour seen in mice (*Mus musculus*) conditioned to expect an aversive foot shock (8).

It is therefore unsurprising that CBD has been hailed by many as a “miracle cure” and has increased in popularity across the world (9). CBD-infused pet products have also become increasingly popular, with pet owners seeking alternative treatment options to support their pets’ physical and emotional health (1, 10, 11). While cannabis-derived veterinary products cannot currently be prescribed within the USA and have variable approval worldwide (11), the market for pet products containing CBD is gaining popularity (12). However, at present there is a lack of regulation around these types of products, which could pose safety risks to both the physical and emotional wellbeing of pets. This highlights the need for evidence-based research into the effects of CBD in companion animal species.

Clinical trials exploring the safety and effectiveness of phytocannabinoids such as CBD in companion animals are now beginning to emerge (10), despite the slow start likely caused by the controversial nature of recreational cannabis use. Current studies have focused primarily on the pharmacokinetics and safety of CBD in dogs (*Canis lupus familiaris*) and more recently cats (13–19), with only a few generating evidence to support the efficacy of CBD in pets. For example, Gamble et al. (14) observed significantly decreased pain and increased activity scores in lame dogs receiving a 2 mg/kg bodyweight (BW) dose of CBD oil every 12 h for 4 weeks. More recently, a study exploring the impact of a single 4 mg/kg BW dose of CBD on acute canine stress observed a mitigating effect of CBD upon multiple stress-related parameters when dogs were exposed to either social separation or car travel (20, 21).

Compared to the somewhat limited research exploring the effect of CBD in dogs, the effect of CBD in cats has received even less attention. Both acute and chronic stress can result in cats displaying fearful and compulsive behaviours, such as aggression, hiding, over-grooming, and inappropriate elimination (22). Such behaviours can often lead to a break down in the human-animal bond, with some owners relinquishing their pet (23). The provision of products containing CBD may therefore offer a solution to some of these issues, reducing the stress response of cats in such situations and thereby mitigating the performance of undesirable behaviours.

Some historical research has explored the influence of ocularly dosed CBD in cats (24, 25) but, to the best of the authors’ knowledge, only four recent studies exploring the effect of CBD in healthy cats have been reported (15, 26–28). These studies primarily focused on the pharmacokinetics of CBD and relied on relatively small sample sizes. Furthermore, these small, homogenous groups of healthy cats are unlikely to have been fully representative of the general pet cat population, making it difficult to generalise the results of these studies.

To address the lack of research surrounding CBD usage in cats, a recent study (19) evaluated the effects a daily 4 mg/kg BW dose of CBD, provided as a THC-free CBD distillate, had on healthy adult cats over a 26 week period, to monitor long term safety of CBD supplementation. It was observed that healthy cats responded well to the long-term administration of CBD, with no lasting health issues being reported. In order to expand upon this, the current study aimed to explore the effect a single 4 mg/kg BW oral dose of CBD had on physiological and behavioural metrics of stress displayed by cats during a subsequent stress-inducing event. Previous studies have demonstrated both the physiological and behavioural impacts of stressors can be quantified using measures such as serum cortisol, body temperature, heart rate, and latency to approach an unfamiliar human (29–33).

Therefore, the first aim of this study was to understand the impact of travel in a cat carrier and introduction to a novel person in an unfamiliar environment (referred to collectively as the stress paradigm) on measures of feline stress. Secondly, this study aimed to evaluate the effect a single dose of CBD (THC-free CBD distillate) had on mitigating stress in cats. It was hypothesised that the selected stress paradigm would induce significant changes in behavioural and physiological indicators of feline stress and that a single dose of CBD would be effective in alleviating these changes in cats, as has previously been observed in dogs (20).

## Materials and methods

### Animals and husbandry

Forty healthy, adult, domestic short hair cats (18 spayed females and 22 neutered males) ranging in age from 1.7 to 12.0 years old ( $\bar{x}$ = 7.1 years old) were selected to participate in this study, based on an *a priori* power calculation. All cats were housed in indoor rooms containing between 5 and 10 individuals (based on group dynamics and the lifestyle identified as most appropriate for those cats) at the Waltham Petcare Science Institute (Leicestershire, United Kingdom). Cats were housed in rooms that are approved and inspected by the UK Home Office under the Animals (Scientific Procedures) Act 1986. Prior to this study, cats were habituated to all sampling equipment, were trained to enter a carrier on cue, and underwent appropriate training to facilitate blood sample collection. In addition, cats were habituated to the rooms used for pre- and post-sample collection prior to study commencement, which required transportation in the trolley-mounted carrier utilized in this study. Four weeks before testing, all cats used in this study were transitioned to the same standard background diet (Royal Canin Instinctive wet and dry food; Royal Canin, St Denis, France, Mars Incorporated) to minimize any potential variation in CBD absorption. Cats were weighed within a week of their treatment to establish an appropriate dose of CBD relative to individual bodyweight. Cats did not participate in any additional studies during this time. This study was approved by the Waltham Animal Welfare and Ethical Review Body (AWERB: WAL 97671) which included two external and entirely independent panel members who are internationally recognised veterinary specialists in animal welfare and ethics.

### CBD and placebo supplements and provision

The hemp-derived CBD distillate and placebo oil utilised in this study were acquired from Kazmira LLC (Colorado, United States). The hemp-derived distillate was diluted with a food-grade sunflower oil and flavoured with 1% rotisserie chicken type natural flavour blend (Apex flavors, Inc. Maryland, United States), to produce a CBD oil with a final CBD concentration of 43.76 mg/mL. This CBD oil was analysed by a third-party laboratory for full spectrum analysis of cannabinoid content (including CBD and THC), potential contaminants, and potency (Botanacor Laboratories, Colorado, United States). The THC content was below the limit of analytical detection (<0.02 mg/mL) and no other cannabinoids were detected except for trace amounts of cannabidivarin (estimated at 0.17 mg/mL), which were below the limit of quantification (0.32 mg/mL). The placebo oil consisted of the same food-grade sunflower oil with 1% rotisserie chicken type natural flavour blend (Apex flavors, Inc. Maryland, United States).

At least one month prior to data collection, cats were screened for CBD palatability to ensure successful consumption during the main study. Cats received a single dose of CBD oil (4 mg/kg BW dose of the CBD oil described above; hereafter referred to as the CBD treatment) mixed with Sheba Creamy Snacks (chicken flavour; Mars Incorporated, Verden, Germany) which was offered at their normal feeding time in a separate bowl from their standard feed. Thirty-seven cats successfully consumed the CBD oil mixed with the Sheba Creamy Snacks on their first exposure. However, three cats refused the CBD treatment. Alternative administration strategies were therefore explored for these individuals. One cat consumed the CBD oil when it was mixed with 8 g of Royal Canin Instinctive wet food (Mars Incorporated, St Denis, France), while another consumed it mixed into 8 g of Sheba Creamy Tuna soup (Mars incorporated, Pak Chong, Thailand). The remaining cat required oral dosing of CBD oil via syringe with an additional 8 g Royal Canin Instinct wet food being provided immediately after. During the main trial eight cats refused to consume the treatment during their second exposure to the stress paradigm. Therefore, these cats were orally dosed with either the CBD oil (four cats) or the placebo oil (four cats), depending on their remaining treatment assignment, via syringe with an additional 8 g Royal Canin Instinct wet food being provided immediately after.

### Study design

**Figure 1:**
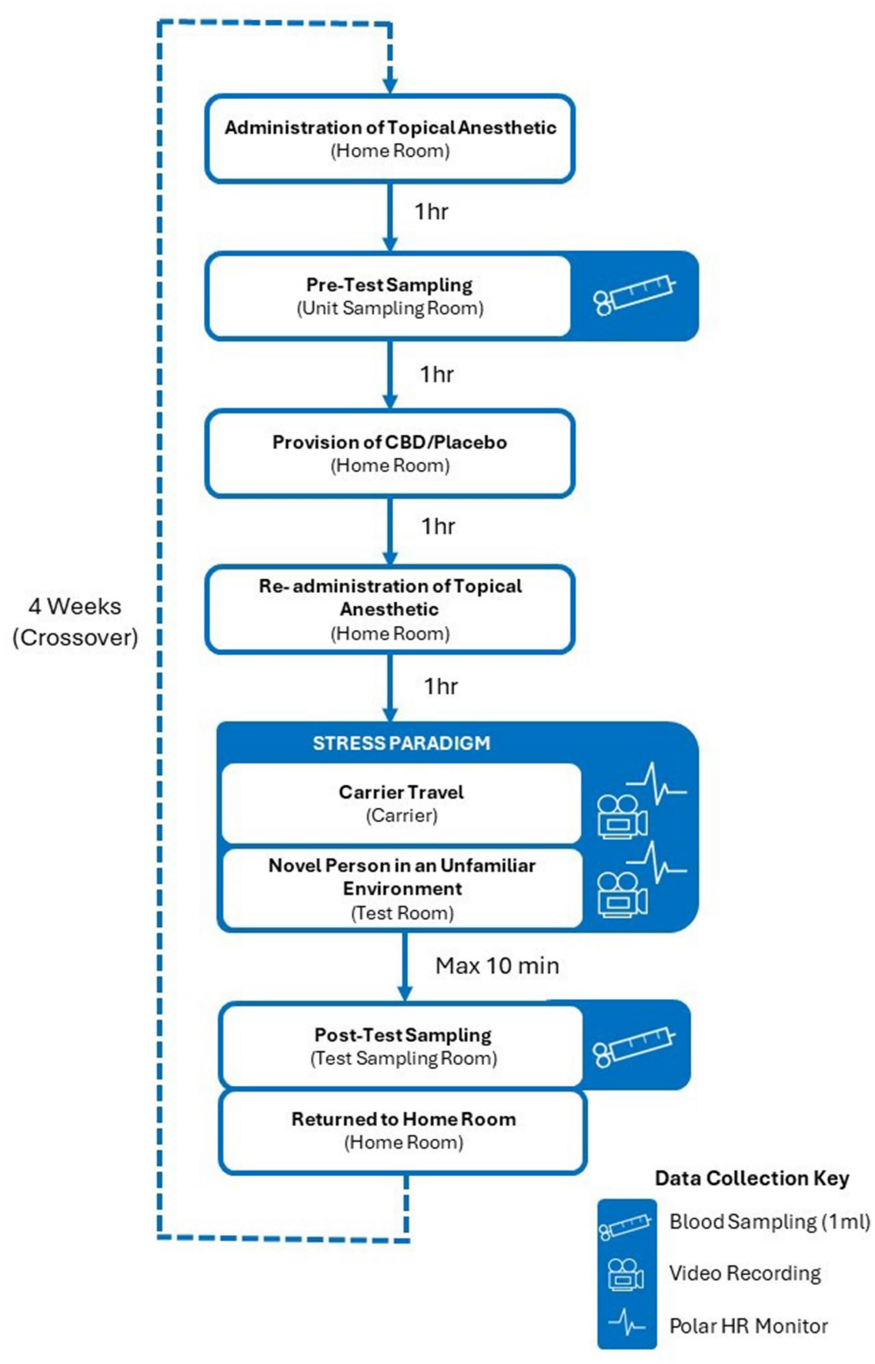
Experimental design. The order in which cats received the CBD treatment was randomized and blinded. Cats that received the CBD treatment prior to their first experience of the stress paradigm received the placebo treatment 4 weeks later, prior to their second exposure. Similarly, cats that received the placebo treatment prior to their first experience of the stress paradigm received the CBD treatment four weeks later, prior to their second exposure.

**Figure 2:**
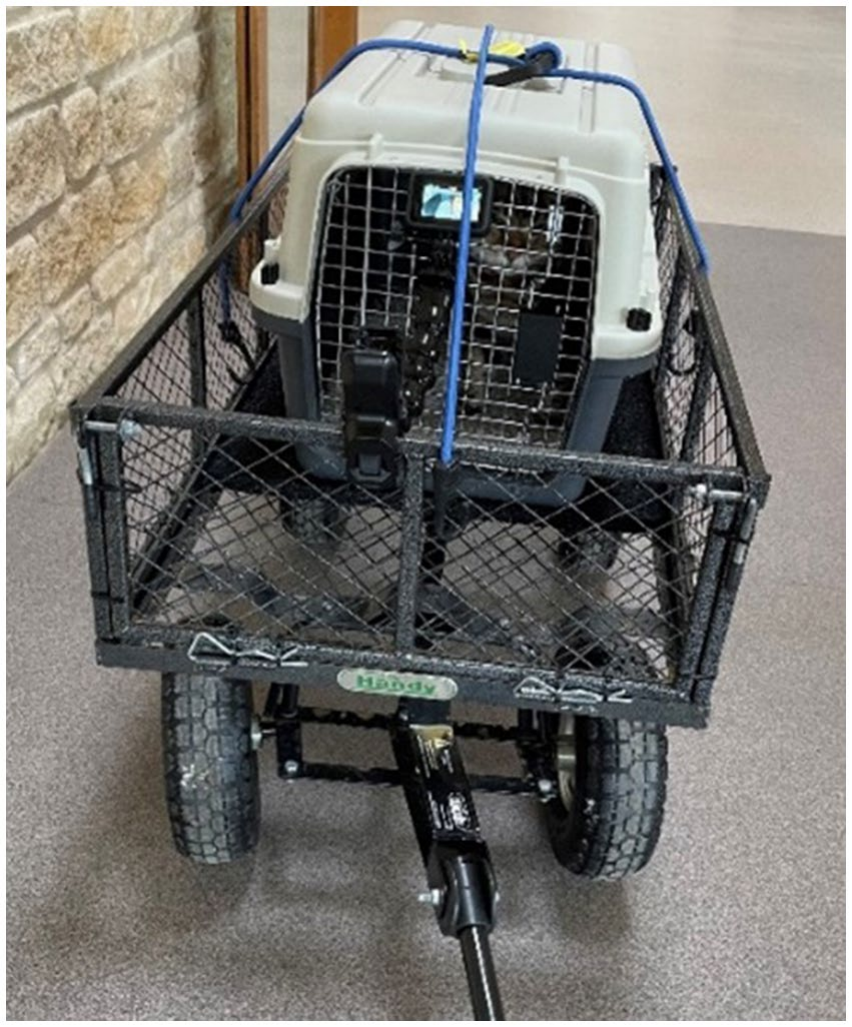
A photo of the cat carrier mounted within the trolley using two crossed bungy cables utilised during the “carrier travel” portion of the stress paradigm.

**Figure 3:**
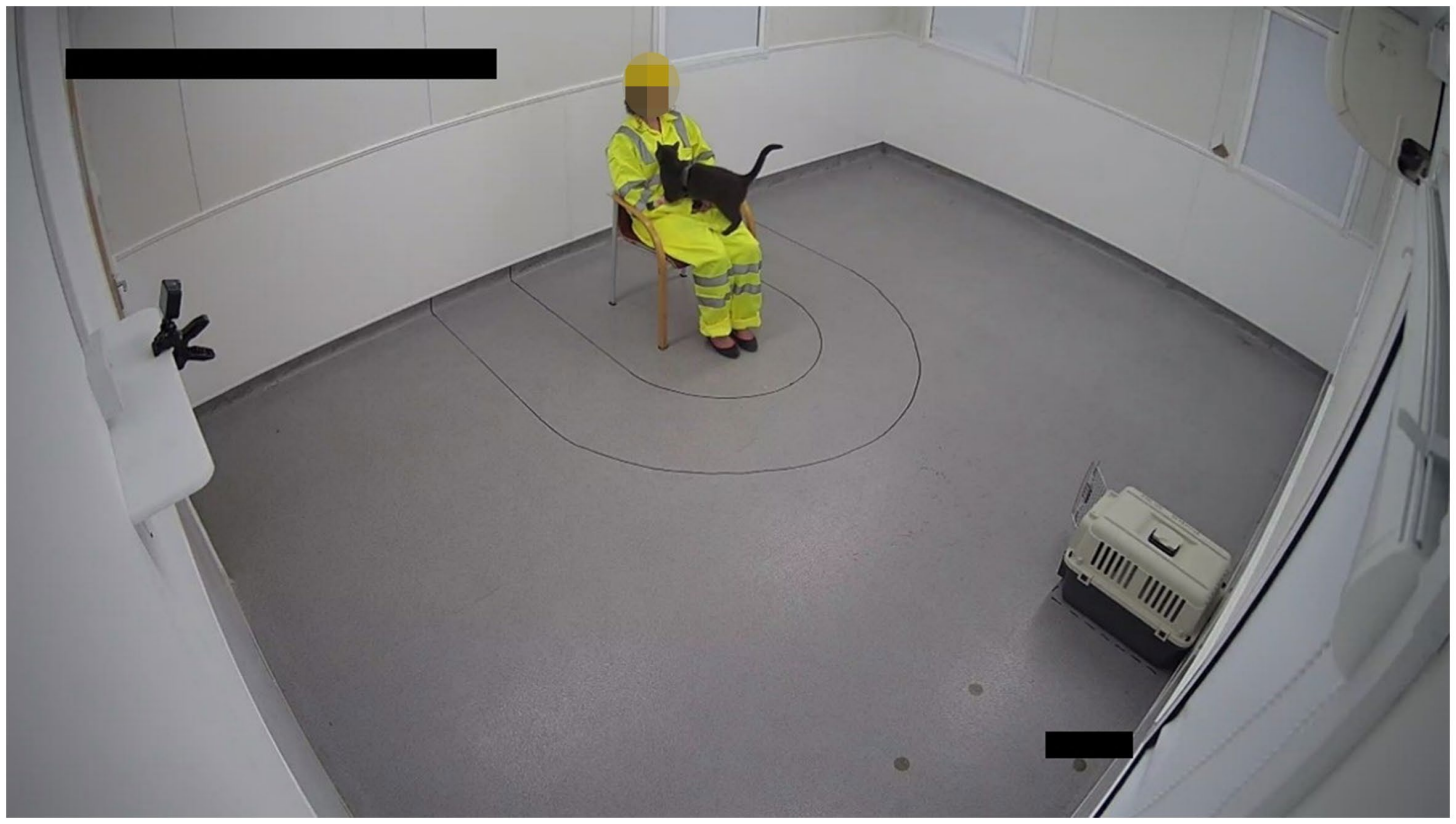
A photo of the testing room showing the novel person, 0.5 m proximity line, 1 m proximity line, the cat carrier, and a cat during the “novel person in an unfamiliar test environment” portion of the stress paradigm.

The main study took place over an eight-week period from May to August 2022 and utilized a balanced, blinded, and randomized crossover design (Figure 1) in which all cats experienced a stress paradigm consisting of travel in a cat carrier mounted on a trolley (Figure 2) and introduction to a novel person in an unfamiliar test environment (Figure 3), on two separate occasions. This crossover design allowed each cat to serve as its own control. Exposure to the stress paradigm occurred four weeks apart, once after consuming a single 4 mg/kg BW dose of CBD and once after consuming a placebo (administered in the same manner as the CBD treatment). Treatments were administered two hours prior to test sessions, based on established pharmokinetics indicating peak mean CBD plasma concentration two hours after ingestion (19). The order in which the CBD and placebo treatments were administered to the cats was randomly assigned and blinding was applied to everyone involved in the trial until after data analysis was complete. Prior to the beginning of the study, a researcher from another department was asked to label the CBD treatment as either Y or Z, with the remaining label being assigned to the placebo. This researcher then revealed which treatment contained CBD upon completion of the analysis.

Various physiological and behavioural measures of feline stress were collected via wearable technology, video cameras, and blood sampling prior to, during, and directly after the test sessions. Cats were monitored via video throughout each test session for signs of distress and/or compromised welfare, based upon predefined removal criteria. No cats that were successfully sampled during the pre-test sampling, and therefore went on to experience the stress paradigm, needed to be removed from the stress paradigm at any point.

### Pre-test sampling

**Figure 4:**
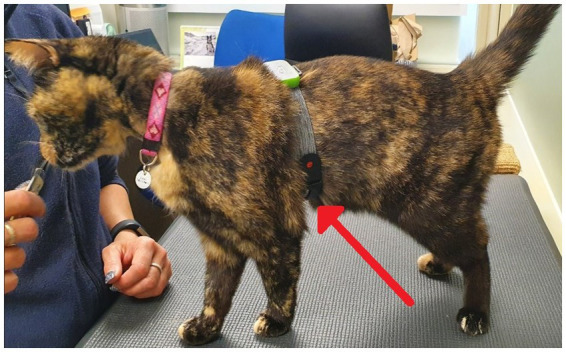
A photo demonstrating the position of the polar heart rate monitor (indicated by red arrow) on cat’s chest as applied using the arm band provided with the monitor.

Prior to collection of pre-testing samples, a small patch of hair was shaved from the blood sampling site (jugular or cephalic vein). Shaving took place within the cats’ home unit sampling room, either the day before, or the morning of, testing. A minimum of one hour prior to sampling, a topical anesthetic (EMLA^™^ cream 5%, AstraZeneca, United Kingdom) was applied to the cats’ skin before it was covered with a wide fabric collar (Kitty Kollar^™^, Garrouzou Inc., United States) for cats sampled from the jugular site, or soft-ban and veterinary wrap for cats sampled from the cephalic vein, to help ensure absorption of EMLA into the skin. Three hours prior to testing, cats were transported to a sampling room on their home unit for pre-test sampling and a 1 mL blood sample was collected from cats to allow for the assessment of baseline measures of serum cortisol, serum Immunoglobulin A (IgA), and serum glucose (with the exception of one individual cat, for whom blood sampling was not attempted throughout the trial due to a history of poor sampling success). The topical anesthetic and covering were reapplied a minimum of 1 hour prior to testing for post-test sampling. Blood samples were attempted but were not obtained for cats during 9 pre-testing sampling situations (3 cats for both treatments, 2 cats during the placebo treatment only, and 1 cat during the CBD treatment only). These cats were returned to their home rooms and were not fed their assigned treatment, nor were they exposed to the subsequent stress paradigm. Successfully sampled cats were returned to their home room. One hour after successful pre-test sampling, cats were fed either the CBD or placebo treatment alongside their standardized diet. Two hours after consumption, cats were equipped with an optical Polar heart rate monitor (Polar^®^ Verity Sense Monitors, Polar Electro Oy, Kempele, Finland) to allow for the continuous measurement of heart rate (HR) and heart rate variability (HRV) throughout the stress paradigm. Polar heart rate monitors were secured around the cat’s chest using the armband provided (Figure 4).

### Stress paradigm

Two hours after the CBD/placebo treatment was administered (i.e., immediately after the cats were equipped with the Polar heart rate monitor), cats were transported from their home unit sampling room to the novel testing room within a standard plastic cat carrier (W:40 cm × D:61 cm × H:41 cm) containing a small piece of non-slip vet bedding, which was mounted securely on a small trolley. Cats were cued to enter the carrier voluntarily, however if a cat did not respond to the cue, they were picked up in a standardized manner and placed in the carrier by a familiar person. Upon entry to the carrier, the door was shut, and the carrier lifted onto the trolley. It was then secured in place using two crossed bungee cables. A GoPro camera (GoPro Hero 7, San Mateo, CA, United States) was mounted to the front of the trolley, pointing towards the front of the carrier in order to capture video footage of the cat. The travel journey took approximately 6 min and followed one of three standardized routes (including outdoor movement between buildings) depending on the location of the cats’ home unit sampling room.

The test room was unfamiliar to all cats and was fitted with a temporarily mounted GoPro camera (GoPro Hero 9, San Mateo, CA, United States) and a permanently installed overhead camera (ELP-USBFHD01M, Ailipu Technology Inc., Shenzen, China). Prior to testing, one of 17 novel people (1 male, 16 female), dressed in fluorescent yellow high visibility overalls and a yellow hard hat, sat on a chair within the room. Markings denoting 1 m and 0.5 m distance from the chair were applied to the floor. During testing, the room temperature was maintained at 19 ± 2°C. On arrival at the testing location, the cat carrier was lifted by a handler from the trolley and placed 2 m directly in front of the novel person. Once the handler exited the room the novel person stood up, walked over to the cat carrier, opened the door, and immediately returned to a seated position on the chair. Cats were able to move freely around the test room for 5 minutes. After 5 minutes, the novel person crouched down in front of the chair, facing the cat and encouraged the cat to approach them (if the cat was not already in contact with the novel person). To encourage the cat, the novel person outstretched their hand toward the cat and said, (“Hi<*cats name*>”). If the cat was already within reach or approached the novel person, the person stroked the cat from head to tail three times during the remaining 30 s of the test. If the cat did not approach, or if the cat remained within the carrier, the novel person stayed in the crouched position for the remaining 30 s and continued to encourage the cat to approach. After 30 s, the handler re-entered the room and the cat was cued to return to the carrier if they were not already inside. If the cat did not respond to the cue, the handler would pick the cat up and place them in the carrier. Cats were subsequently transported to an adjacent sampling room for collection of post-test samples.

### Post-testing sampling

An additional 1 mL blood sample was collected a maximum of 10 min after the completion of the stress paradigm. High value rewards (including canned tuna and catnip) were given to the cats after post-test sampling, to minimize the formation of negative associations. Cats were then returned to their home unit using the trolley-mounted carrier following the most direct route possible.

### Physiological and behavioural measures

#### Serum cortisol, immunoglobulin A, and glucose

A total of 70 pre-testing blood samples were collected (CBD treatment = 36, placebo treatment = 34), while a total of 61 post-testing blood samples were collected (CBD treatment = 29, placebo treatment = 32). All four test samples were successfully collected for 26 cats. Blood samples were collected into a clot-activating serum tube and kept on ice for a maximum of 60 min before being aliquoted for IgA, cortisol, and glucose analysis. Aliquots for cortisol and IgA were stored at −20°C in preparation for later analyses. Serum cortisol was examined using the R&D Systems, Parameter^™^ cortisol immunoassay (bio-techne, Minneapolis, United States) as per the manufacturer’s protocol with an intra-assay variation of <10%. Analysis of IgA was conducted using an Abcam, IgA Cat ELISA kit (Boston, United States), following the protocol provided by the manufacturer. Serum glucose was quantified on an AU480 Clinical Chemistry Analyser (Beckman Coulter, High Wycombe, United Kingdom) using Beckman Coulter Glucose reagents (OSR6121), serum calibrators (66300), and QC material (ODC0003 & ODC0004) as per the manufacturer’s instructions. Samples were bracketed by pre- and post-analysis quality controls, providing an intra-assay variation of <2 × standard deviation. Two pre-testing CBD treatment samples could not be analyzed due to incomplete samples being obtained. Additionally, one pre-testing CBD treatment sample and one pre-testing placebo treatment sample could only be analyzed for IgA, due to limited blood volume.

#### Heart rate, heart rate variability

Cats were fitted with a polar heart rate monitor in order to capture heart rate (HR) via optical technology during the “carrier travel” and “novel person in an unfamiliar environment” portions of the paradigm. HR data were later converted to heart rate variability (HRV), which was calculated as the root mean square of successive differences between normal heartbeats (RMSSD).

#### Cat stress score

Video footage collected from the GoPro attached to the front of the cat carrier during transportation was analyzed by two trained raters who were familiar with cat behaviour and were blinded to the treatment received. Cat stress scores (CSS; (34)) were obtained at 30 s intervals for all recorded sessions. The CSS scale enables observers to score a cat’s body language between 1 (fully relaxed) and 7 (terrified), focusing on each of the following observable body areas: body, stomach, legs, tail, head, eyes, pupils, ears, and whiskers, as well as the presence of vocalizations and full body activity levels (Supplementary Table S1). Eleven instantaneous CSS scores were generated per video and were averaged to produce an overall mean CSS score between 1 and 7 per individual for each of the two coders. Coders were also asked to re-score five randomly selected videos twice more in order to calculate intra-rater reliability.

#### Qualitative behaviour assessment

During the “novel person in an unfamiliar environment” portion of the stress paradigm, a series of cat behaviour attributes were assessed using qualitative behavioural assessment (QBA). The QBA was developed and modified using terms gathered from QBAs utilized in previous studies evaluating welfare in shelter dogs (35), and dogs during car travel (20, 36). Additional terms (“Affectionate”, “Confident”, “Friendly”, “Frustrated”, and “Suspicious”) were included to meet the requirements of the present study. A final set of 23 terms were used to score cats’ behavioural response to being in an unfamiliar room with a novel person (Table 1). Three blinded and trained raters who were familiar with cat behaviour scored QBAs using the videos recorded by the GoPro temporarily mounted in the testing room.

**Table 1:** Terms included in the QBA to evaluate cat behaviour during the “novel person in an unfamiliar test environment” portion of the stress paradigm’.

Term	Definition
Affectionate	Seeking tactile engagement with novel person
Aggressive	Exhibits aggressive body language, e.g., growling, hissing
Alert	Vigilant, inquisitive, on guard
Anxious	Worried, unable to settle or cope with the environment, apprehensive
Bored	Disinterested, passive, showing sub-optimal arousal levels/drowsiness signs
Calm	Absent of strong positive/negative emotions
Confident	Assertive, absence of negative emotions, purposeful movement
Comfortable	Without worries, settled in environment, peaceful with external stimuli
Curious	Actively interested in people or things, explorative, inquiring, in a positive relaxed manner
Depressed	Dull, sad demeanour, disengaged from and unresponsive to the environment, quiet, apathetic
Explorative	Confident in exploring the environment or new stimuli, investigative
Fearful	Timid, scared, shows postures typical of fear
Friendly	Benevolent, comfortable around novel person, exhibits affiliative body language
Frustrated	Negative emotional state due to reduction in autonomy or due to novel person disengagement
Interested	Attentive, attracted to stimuli and attempting to approach them
Nervous	Uneasy, agitated, shows fast arousal, unsettled, restless, hyperactive
Reactive	Responsive to external stimuli
Relaxed	Easy going, calm with no visual evidence of tension in the body
Restless	Unable to rest of relax, pacing
Sad	Unhappy, downcast
Stressed	Tense, shows signs of distress
Suspicious	Unsure, doubtful, shows conflicting behaviour, uncertain whether to approach or trust a stimulus
Tense	Stiff, rigid positive, on edge

Each rater completed an online form for each video, which comprised a Visual Analogue Scale (VAS) ranging from 0 to 125 placed next to each term. The left end of the VAS scale corresponded to the minimum score possible (0), which represented the expressive quality indicated by the term being entirely absent in the cat. On the other hand, the right end of the scale represented a maximum score (125), meaning that the quality indicated by the term was strongly present in that cat. Raters were instructed to watch the videos and select a point along the VAS which they felt was appropriate for each term immediately after the video had finished. The numerical values associated with their selected point on the scale were not visible to the rater. Additionally, five videos were selected at random and re-watched twice by all observers to calculate intra-rater reliability.

#### Additional coded behaviours

Cat behaviour, including latency to approach the novel person during the “novel person in an unfamiliar environment” portion of the stress paradigm, was coded by two trained raters using videos collected by the video cameras mounted in the test room. Each coder annotated specific cat behaviours (Table 2) from footage using “The Observer XT 15” software.

**Table 2:** Ethogram used to measure cat behaviour during the “novel person in an unfamiliar test environment” portion of the stress paradigm’.

Term	Type		Definition
Inside of carrier	Event		The cat is fully inside of the carrier or has 1 or more paws inside the carrier
Outside of carrier	Event		The cat is outside of the carrier with all 4 paws
Approach to 0.5 m	Event		Any two paws of the cat have crossed the 0.5 m line
Approach to 1 m	Event		Any two paws of the cat have crossed the 1.0 m line
Contact with novel person	State	Start	The cat is touching the novel person with any part of their body
		Finish	Behaviour ceases, the cat is not touching the novel person with any part of their body
Vocalisation	Event		The cat is emitting sound from the mouth
Touching with novel person while receiving encouragement	State	Start	The novel person has stood up off the chair and encouraged the cat over, the cat is making contact with the novel person with any part of its body
		Finish	The novel person has stood up off the chair and encouraged the cat over, the cat is not making contact with the person with any part of its body

Behavioural coding of videos began from the moment the novel person opened the carrier and ended 5 minutes and 30 s later. To calculate inter-rater reliability, nine randomly selected videos were coded by both raters. To calculate intra-rater reliability, each rater re-coded five randomly selected videos two more times.

### Statistical analyses

All analyses were performed in R version 4.2.2 (The R Foundation for Statistical Computing). The inter- and intra-rater reliability of CSSs, QBA scores, and behavioural coding was assessed by interclass correlation coefficients (ICCs) using two-way mixed effects models. Consistency agreement was used for inter-rater reliability, and absolute agreement was used for intra-rater reliability. ICC ratings were considered to be poor, moderate, good, or excellent based on the guidelines suggested by Koo and Li ((37); Excellent: ICC > 0.90, Good: 0.75 < ICC < 0.90, Moderate: 0.50 < ICC < 0.75, Poor: ICC < 0.5).

All measures, with the exception of individual term QBA scores, (i.e., cortisol, IgA, glucose, mean HRV, mean and maximum HR, mean CSS, duration of vocalisations performed, duration of time spent in the carrier, duration of time spent in contact with the novel person while not receiving encouragement, duration of time spent touching the novel person while receiving encouragement, latency to approach 1.0 m, and latency to approach 0.5 m) were evaluated using linear mixed effects modelling (LMEM). All models included a fixed effect for treatment and a random effect for cat (intercept-only). Models for physiological measures included additional fixed effects; in the cases of cortisol, IgA, and glucose for sampling timepoint (i.e., pre- vs. post-test), plus the two-way interaction between treatment and timepoint, and in the case of polar heart rate measures (HR and HRV), for the portion of paradigm being recorded (i.e., “carrier travel” vs. “novel person in an unfamiliar test environment”), plus the two-way interaction between the portion of the paradigm and treatment. Models for CSS and additional coded behaviours included no additional effects.

For measures collected only at single timepoints (i.e., behavioural measures), planned pairwise comparisons were conducted between treatment groups. For measures collected at multiple timepoints (i.e., physiological measures, HR and HRV), planned pairwise comparisons were conducted between treatment groups for each timepoint, and between timepoints for each treatment group. Reported *p*-values for pairwise differences and 95% confidence intervals for mean estimates were FWE corrected (i.e., for multiple comparisons) within, but not across, models for each outcome variable using the glht function’s default “single-step” approach.

Due to untransformed model residuals severely violating the normality assumption, models for several variables (i.e., IgA, mean HRV, duration of vocalisations, and duration of time in contact with stranger) were conducted on log10 transformed data. In these cases, reported mean estimates were back-transformed and differences were reported as fold-changes (i.e., ratios). QBA scores were analysed using an exploratory multivariate approach, fitting a principal component analysis (PCA) to the data and then plotting by treatment condition and by animal to visualise any differences. The two best fitted primary components (PC1 and PC2; described below) were extracted and additionally modelled using the same LMEM method described above.

## Results

### Serum cortisol, IgA, and glucose

**Figure 5:**
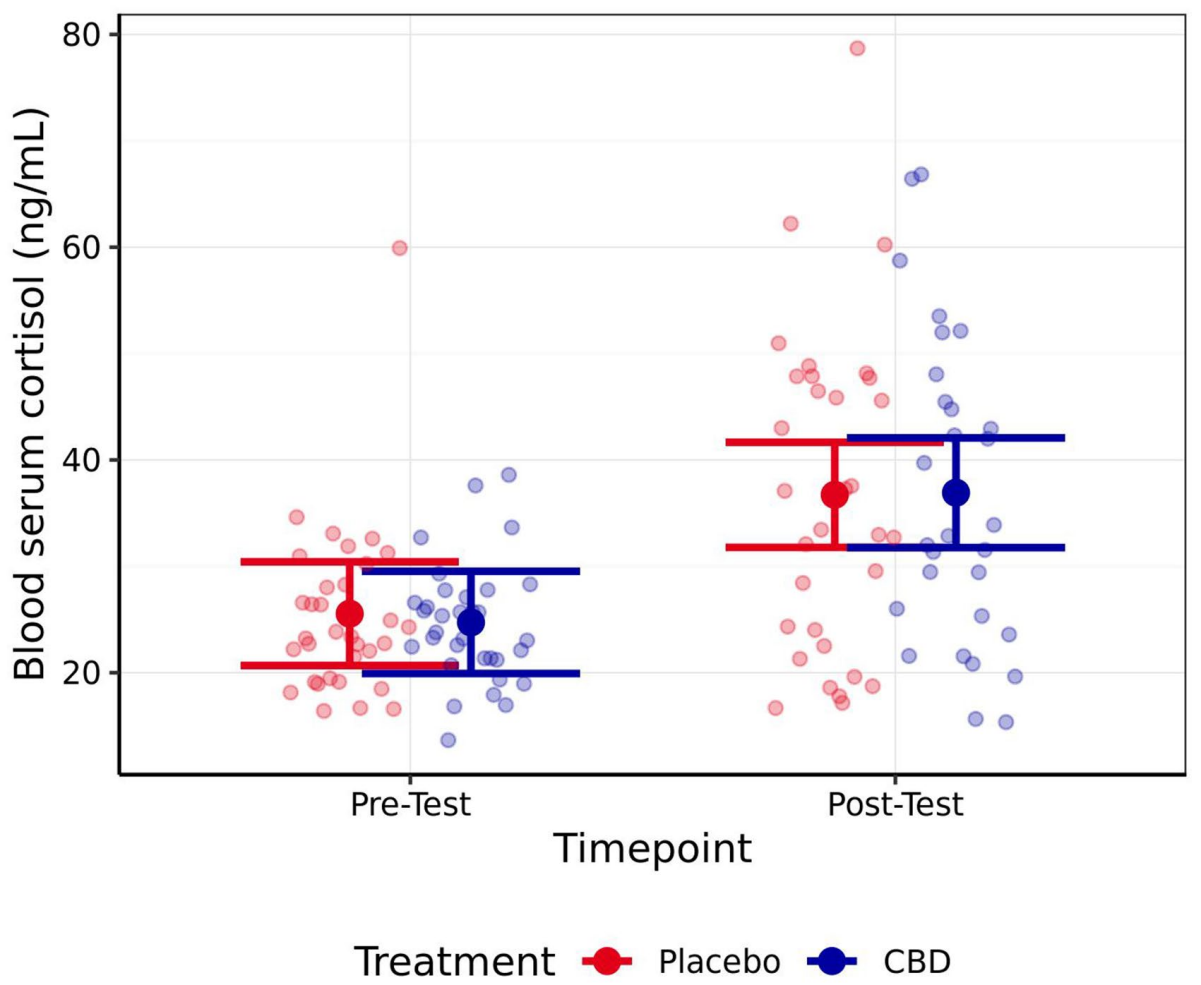
Mean estimate serum cortisol levels pre- and post-testing for both the CBD and Placebo treatments. Error bars represent 95% confidence intervals (FWE-adjusted). Number of observations = 128.

No significant difference in cortisol was observed between the CBD and placebo treatment at either the pre-test (*z* = 0.34, *p* = 0.98) or post-test (*z* = −0.08, *p* > 0.99) timepoint (Figure 5). However, cats demonstrated significantly higher cortisol levels during post-testing samples compared to pre-testing samples, regardless of treatment (CBD *z* = 4.93, *p* < 0.001; Placebo *z* = 4.64, *p* < 0.001). No significant differences were observed between pre- or post-testing samples in either serum IgA (CBD *z* = 1.09, *p* = 0.64; Placebo *z* = −0.10, *p* > 0.99) or glucose (CBD *z* = −0.07, *p* > 0.99; Placebo *z* = −1.39, *p* = 0.44). Additionally, no significant differences between treatments were observed for these measures, at either timepoint (IgA: Baseline *z* = 1.61, *p* = 0.31; Post-test *z* = 0.35, *p* = 0.98; Glucose: Baseline *z* = 1.10, *p* = 0.64; Post-test *z* = −0.21, *p* > 0.99). Mean estimates for serum cortisol, IgA, and glucose are presented in Table 3.

**Table 3:** Mean estimate (ME) serum cortisol (ng/mL), immunoglobulin A (IgA) (ng/mL), and glucose (mmol/l) for the CBD and placebo treatment collected during both pre- and post-test sampling.

	Treatment											
	CBD						Placebo					
	Pre-test			Post-test			Pre-test			Post-test		
	ME	Upper CI	Lower CI	ME	Upper CI	Lower CI	ME	Upper CI	Lower CI	ME	Upper CI	Lower CI
Serum Cortisol	24.73^a^	29.54	19.92	36.92^a^	42.08	31.76	25.55^b^	30.42	20.67	36.72^b^	41.66	31.78
IgA	1.027	1.546	682.2	1.079	1.626	715.6	1.102	1.659	731.5	1.097	1.652	727.9
Glucose	4.47	4.67	4.27	2.46	4.67	4.26	4.57	4.77	4.37	4.44	4.64	4.24

Corresponding superscript letters indicate pairs of MEs that significantly differed between treatments or sampling times. Upper and lower confidence intervals (CI) are also presented.

### Heart rate and heart rate variability

**Figure 6:**
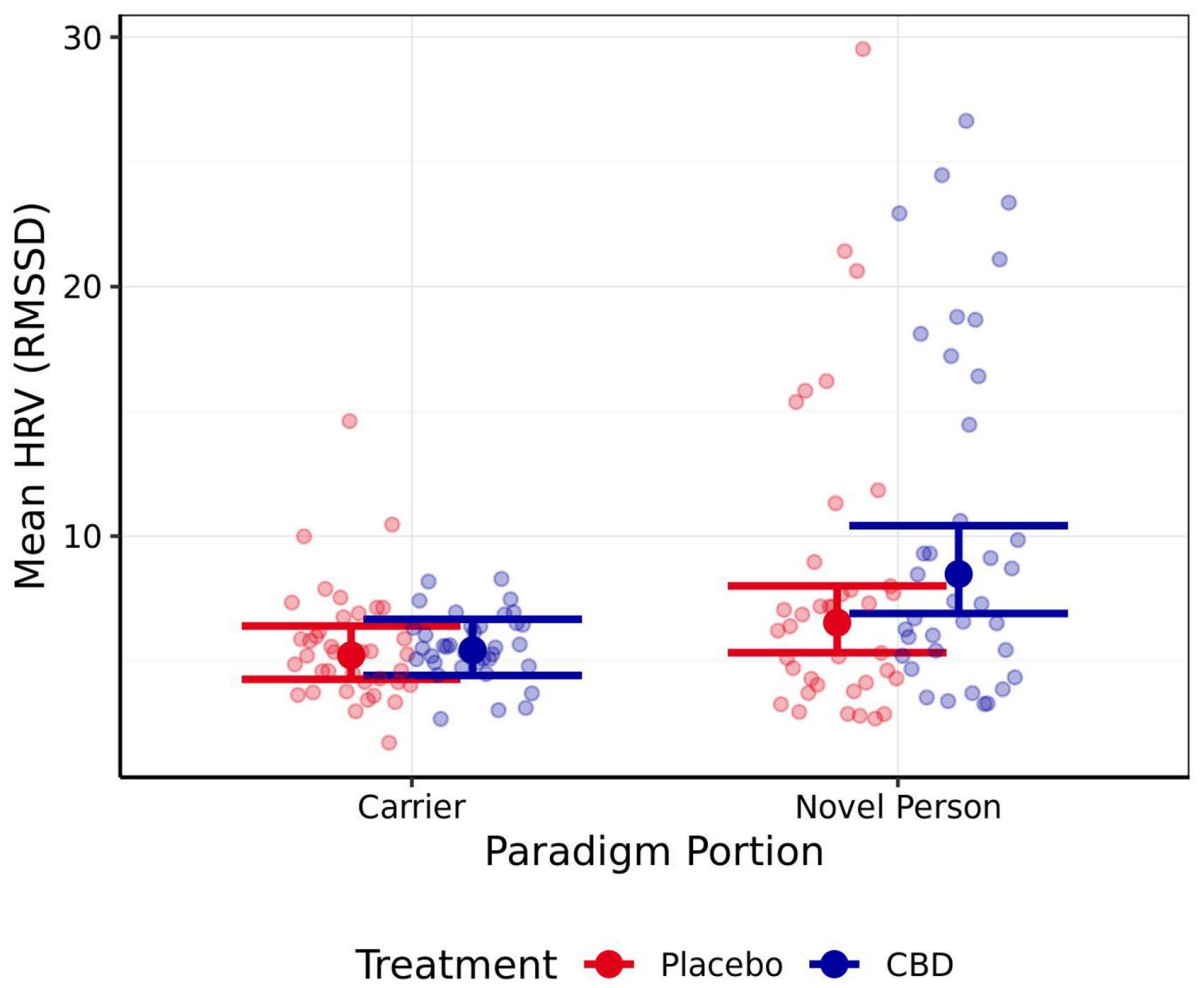
Mean estimate heart rate variability (HRV) for both the CBD and Placebo treatments during the “carrier travel” (carrier) portion and the “novel person in an unfamiliar test environment” (novel person) portion of the stress paradigm (back transformed). Error bars represent 95% confidence intervals (FWE-adjusted). Number of observations = 150.

Neither mean nor maximum heart rate differed significantly between either portion of the stress paradigm, or the treatment provided (all |*z*| < 1.35, *p* > 0.40; mean estimates are presented in Table 4). Mean HRV was significantly higher for the “novel person in an unfamiliar environment” than the “carrier travel” portion of the stress paradigm for the CBD treatment, but not the placebo treatment (CBD *z* = 4.01, *p* < 0.001; Placebo *z* = 2.04, *p* = 0.14; Figure 6). The differences in mean HRV between the CBD and placebo treatment were non-significant for each portion of the paradigm (both *p* > 0.05), but a trend was evident in the “novel person in an unfamiliar environment” portion of the paradigm for higher HRV following CBD treatment compared to the placebo (*z* = −2.36, *p* = 0.06; representing a 23% reduction in HRV estimate for placebo vs. CBD treatment).

**Table 4:** Mean estimate (ME) mean and maximum heart rate (HR) for the “carrier travel” (carrier) and “novel person in an unfamiliar environment” (novel person) portions of the stress paradigm (all presented as beats per minute/BPM).

	Treatment											
	CBD						Placebo					
	Carrier			Novel Person			Carrier			Novel Person		
	ME	Upper CI	Lower CI	ME	Upper CI	Lower CI	ME	Upper CI	Lower CI	ME	Upper CI	Lower CI
Mean HR	86.17	92.59	79.74	82.65	89.08	76.23	89.50	95.85	83.15	86.11	92.45	79.76
Maximum HR	99.51^a^	106.10	92.93	103.80^a^	110.40	97.20	102.90	109.40	96.36	101.40	107.90	94.89

Corresponding superscript letters indicate pairs of MEs that significantly differed between treatments or paradigm portions. Upper and lower confidence intervals (CI) are also presented.

### Cat stress score

When exploring the CSS, inter-rater and intra-rater reliability were observed to be good and excellent, respectively. An average CSS score was therefore taken from both coders, to produce an overall average CSS score for each “carrier travel” video, which ranged between 2 (weakly relaxed) and 4.2 (very tense). No significant differences in CSS were evident during the “carrier travel” portion of the paradigm between CBD and placebo treatment (*z* = −0.57, *p* = 0.57; mean estimates are presented in Supplementary Table S2).

### Qualitative behaviour assessment

No QBA data were available for 13 “novel person in an unfamiliar environment” sessions during which the cat failed to leave the carrier and was therefore not visible on the camera. These included both treatment sessions for five cats, only the placebo treatment session for two cats, and only the CBD treatment session for one cat.

Inter-rater reliability for individual QBA terms ranged from poor (“Aggressive”, “Alert”, “Bored”, “Curious”, “Depressed”, “Frustrated”, “Interested”, “Reactive”, “Sad”) to moderate (“Affectionate”, “Anxious”, “Calm”, “Comfortable”, “Confident”, “Explorative”, “Fearful”, “Friendly”, “Nervous”, “Relaxed”, “Restless”, “Stressed”, “Suspicious”, “Tense”). Intra-rater reliability for the individual terms ranged from poor to excellent depending on the term and rater. Based on these results, one rater (Rater 1) was removed from the analysis of the QBA data, and only ratings from the remaining two raters were used for further analysis. Additionally, the terms “Alert” and “Reactive” were removed from the QBA due to poor intra-rater reliability. Inter-rater reliability was repeated for the two remaining raters, revealing an excellent reliability for the terms “Affectionate” and “Suspicious”, a good reliability for the terms “Anxious”, “Confident”, “Comfortable”, “Explorative”, “Friendly”, “Nervous”, “Restless”, “Stressed”, “Tense”, a moderate reliability for the terms “Bored”, “Calm”, “Curious”, “Fearful”, “Frustrated”, “Interested”, “Relaxed”, and a poor reliability for the terms “Aggressive”, “Depressed”, “Sad”.

**Figure 7:**
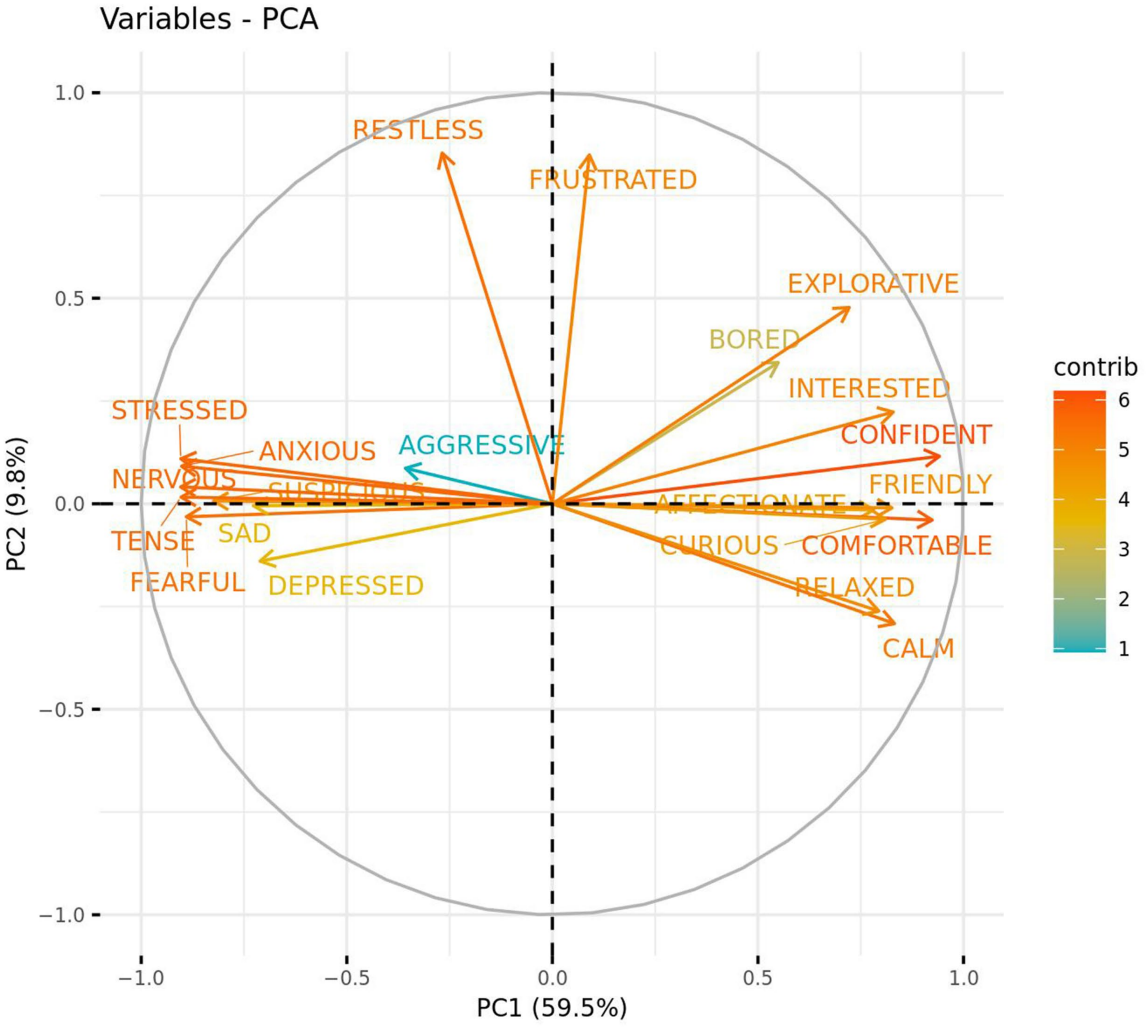
Strength and direction in which qualitative behaviour assessment (QBA) terms loaded against two PCA component scores. PC1 represents positive vs. negative emotions while PC2 represents active vs. passive behaviour. Bracketed numbers represent the total variance explained by each component score.

Two primary components (PC1 and PC2) of the QBA data were extracted through use of a PCA and appeared to represent positive vs. negative emotions (PC1—59.5%), and active vs. passive behaviour (PC2—9.8%). Using a loading cut-off of >|0.50|, QBA scores for “Confident”, “Comfortable”, “Calm”, “Interested”, “Friendly”, “Curious”, “Relaxed”, “Affectionate”, “Explorative”, and “Bored” loaded positively onto PC1 while “Nervous”, “Stressed”, “Tense”, “Anxious”, “Fearful”, “Suspicious”, “Sad”, and “Depressed” loaded negatively. PC2 demonstrated positive loading for both “Restless” and “Frustrated” QBA scores (Figure 7). ICCs were also calculated post-PCA in order to assess inter- and intra-rater reliability of PC1 and PC2. Inter-rater reliability was good for PC1 and PC2. Intra-rater reliability for PC1 was excellent for both remaining coders, and for PC2 was good for Rater 2 and excellent for Rater 3.

**Figure 8:**
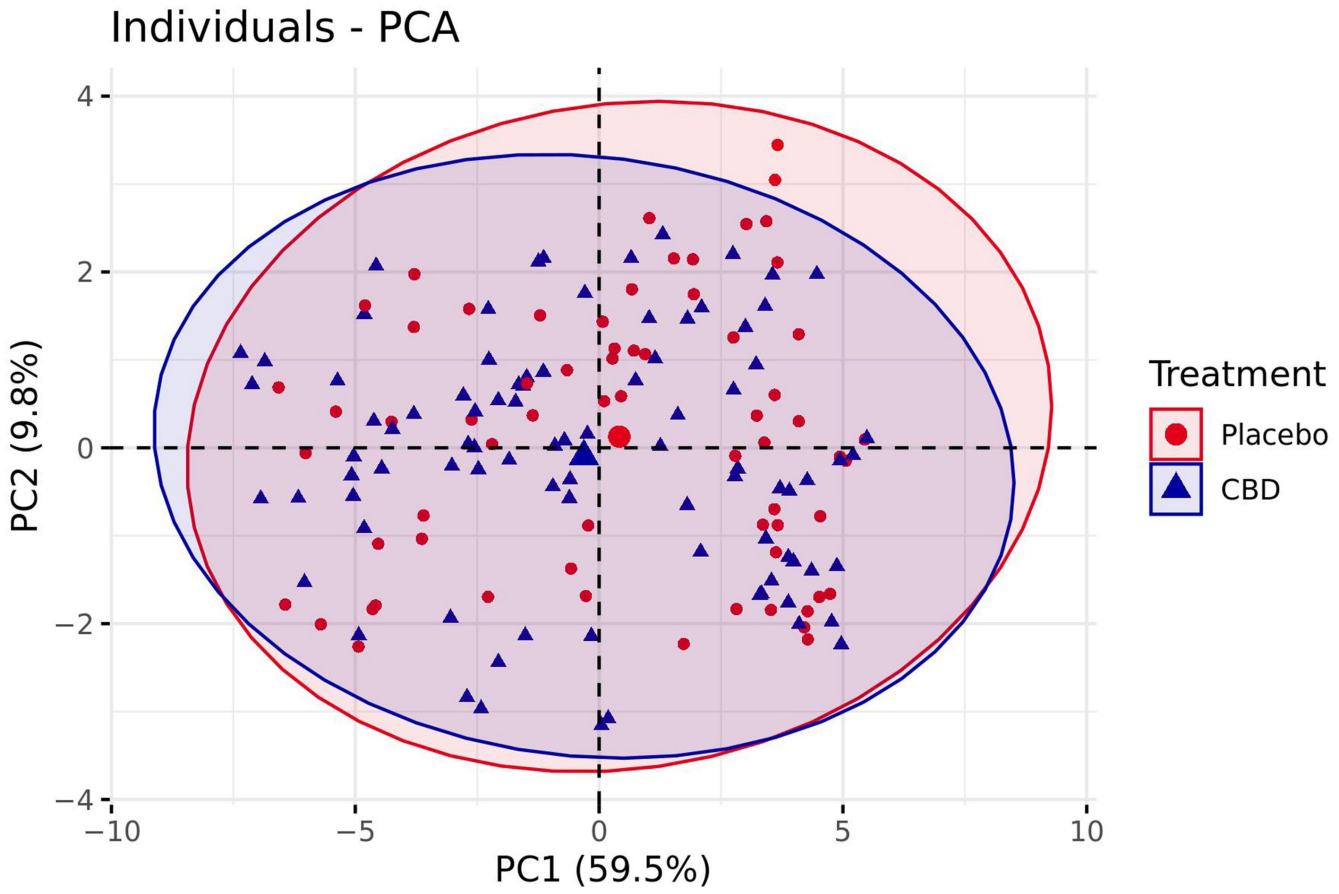
Comparison of PC1 and PC2 scores for both the CBD and Placebo treatments plotted for each individual cat. Bracketed numbers represent the variance explained by each principal component. Number of observations = 67.

Plotting of the individual component scores revealed very little separation between treatment groups, with a negligible bias towards more positive emotion following the placebo treatment, and data ellipses overlapping considerably (Figure 8). Additionally, modelling revealed no significant difference between the placebo and CBD treatment group for PC1 (*z* = −0.98, *p* = 0.33) or PC2 (*z* = −0.22, *p* = 0.83). Mean estimates for PC1 and PC2 are presented in Supplementary Table S2.

### Additional coded behaviours

Inter-rater reliability was excellent for all coded behaviours. Furthermore, intra-rater reliability was excellent for the two coders for all behaviours with the exception of “contact with the novel person”, which was good for one rater. No significant effect of treatment was observed for any of the behaviours coded during the “novel person in an unfamiliar test environment” portion of the stress paradigm (all |*z*| < 1.4, *p* > 0.10; mean estimates are presented in Supplementary Table S2).

## Discussion

The aim of this study was to investigate the impact of providing a single dose of CBD distillate on measures of feline stress when cats were exposed to a sequence of stressful events, i.e., travel in a cat carrier followed by meeting a novel person in an unfamiliar environment. Serum cortisol significantly increased after exposure to the stress paradigm, suggesting that these events were stressful for this population of cats. However, no significant differences in this response were observed when cats were provided with a CBD treatment compared to when they were provided with a placebo. Similarly, no effect of CBD was observed on serum IgA or glucose.

No significant differences in mean or maximum HR were observed between either portion of the stress paradigm. Furthermore, no effect of CBD was observed on HR. However, mean HRV was shown to be significantly higher for the “novel person in an unfamiliar environment” portion than the “carrier travel” portion when cats were provided with the CBD treatment. Reduced HRV has been considered to represent a more negative emotional state in dogs (38), which may suggest that cats provided with CBD had a more positive experience during the “novel person in an unfamiliar test environment” portion of the stress paradigm than they did in the “carrier travel” portion. It should be noted, however, that the estimated means for both mean and maximum HR throughout this study (mean HR; $\bar{x}$ = 86 ± SD 16 beats/min, maximum HR = $\bar{x}$ 102 ± SD 16 beats/min) were comparatively low compared to what is typically expected for cats within a non-stressful home environment, as measured using an electrocardiogram (mean HR: $\bar{x}$ = 132 ± SD19 beats/min; (39)). Because this study required cats to be conscious and fully mobile, the sternum was selected as the optimal location to position the optical Polar heart rate monitors. However, a study exploring the suitability of different body locations for the use of photoplethysmography on anesthetized cats suggested that the tongue was the optimal place for measuring pulse rate, with the sternum resulting in low signal power and kurtosis (40). This may explain why over 25% of the HR readings for 16 of the 150 HR recording sessions were found to be unavailable for analysis. These limitations surrounding the use of optical HR monitor on the sternum suggest that results relating to HR and HRV within this study should be interpreted with caution. Future studies should investigate the suitability and placement of optical HR monitors on cats, explore alternative ways of measuring HR, and/or consider acceptable thresholds for HR data completeness required for valid inclusion in final analyses.

In addition to HR, no significant effect of treatment was observed for any of the behavioural metrics collected. It is possible that the limited effect of CBD on both the behaviour and physiology of cats observed in this study was a result of the dosage provided. Previous research in dogs observed a single 4 mg/kg BW oral dose of CBD was effective in positively influencing some indicators of acute canine stress when dogs were exposed to stress inducing events (20). However, during a study exploring the pharmacokinetics of CBD in dogs and cats, Deabold and colleagues (15) observed that the absorption kinetics of a 2 mg/kg BW CBD dose within cats resulted in a maximal serum concentration of approximately one-fifth of what was observed within dogs (cats = 43 ng/mL SE ± 9; dogs = 302 ng/mL SE ± 63). Cats also appeared to show a longer retention time than dogs (cats = 3.5 ± 1.4 h, dogs = 1.4 ± 0.3 h) and a CBD half-life of 2.4 h. These findings suggest that the absorption of oil-based CBD is lower in cats than dogs and that a larger dosage may be required to induce a similar pharmacological or behavioural effect. However, it should be noted that more recent studies in cats have observed higher average maximum serum CBD concentrations ((26); 236 ng/mL SD ± 193; (27); 282 ng/mL SD ± 149) than those observed by Deabold et al. (15). In agreement with this, Coltherd et al. (19) observed an average maximum serum CBD concentration of 251 ng/mL 95%CL: 108.7–393.4 when utilizing identical CBD dose and delivery matrix to the one utilized in this study. These differences are likely due to variation in dosages, accumulation of CBD in blood plasma over repeated doses, variation in the matrix used for CBD delivery (i.e., MCT oil, fish oil, food-based paste, etc.), and the presence of other cannabinoids in addition to CBD (26–28).

Multiple studies have suggested an “entourage effect” in which additional cannabinoids and other compounds found in cannabis may interact with, and possibly increase absorption of, CBD (41, 42). Additionally, Rozental et al. (28) suggest that due to its nature as a lipid-soluble drug, variation in body condition and the fat content of individual cats may affect the potential for CBD to be held within the body. It is therefore suggested that all future studies exploring the efficacy of CBD in cats should consider monitoring serum CBD concentrations to account for individual variation in CBD absorption.

Inconsistencies in CBD dosing may also have arisen during the current study due to the viscous nature of the CBD oil utilised. Remnants of the CBD oil after feeding were often observed on plates and inside the oral dosing syringe, suggesting that cats may not have received the intended CBD dosage. Furthermore, a previous study observed a negative behavioural response to CBD administration with cats performing head-shaking and excessive licking (15). Kulpa and colleagues (26) suggest that these negative effects may be the result of an immediate aversion to the taste or smell of the CBD oil. While such reactions were not observed during this study, multiple cats refused to voluntarily consume their treatment (both CBD and placebo) prior to their second exposure to the stress paradigm. This may be due the development of a negative association between the provision of the treatment and the taste of CBD itself, the taste of the flavoured sunflower oil used to create both the CBD and placebo treatments, or even the subsequent stress paradigm. While further investigation into the reason for these refusals is required, there is potential for future studies to explore methods of increasing the palatability of CBD for cats.

Variation between individual’s response to the stress paradigm was, in part, accounted for in this study by using a random order cross-over experimental design, in which individual cat was treated as a random effect within the analysis. However, it is possible that variation in individual temperament or coping styles may have impacted the effectiveness of the CBD treatment. It should be noted that a large variation in individual responses to the stress paradigm were observed during this study. It has been suggested that a cat’s response to stress not only depends on the environment in which the cat lives, but also its individual temperament (22). Temperament is typically defined as an individual’s behaviour that is stable across time and situations (43) and is dependent upon both an individual’s genetic make-up and early life experiences (22). For example, some studies have observed an influence of breed and even coat colour on cat sociability (44, 45). Additionally, known personality traits such as “Shy”, “Mellow”, “Timid”, “Playful”, “Active”, and “Curious”, have been found to impact cat response styles in tests exploring latency to approach an unfamiliar person (33). Therefore, it is possible that differences in individuals’ temperament may have induced variation in response to the stress paradigm utilised in this study. Unfortunately, this study was unable to fully consider (and account for) the effect of variation in cats coping styles on CBD effectiveness. The absence of baseline behaviour data for the cats used in this study may have limited our ability to fully explore the impact of CBD on cat behaviour. However, in this instance, such baseline data may not have been informative due to the lack of standardisation and variability in conditions between cats’ home environments and the test room. We recognise the value of measuring behaviour prior to exposure to a stress paradigm and will endeavour to undertake this in relevant future studies.

It should also be noted that while this study did utilise a considerably larger sample size than those that have previously explored the effect of CBD on cat affective states, care should be taken to minimise the generalisation of these results to broader cat populations. All individuals utilised within this study were raised within a research facility and consequently may not have demonstrated temperaments or coping styles representative of the general pet cat population.

Despite these limitations, this study has demonstrated that the combined exposure of cats to “carrier travel” and a “novel person in an unfamiliar environment” was successful in inducing a physiological stress response in cats, as indicated by an increase in serum cortisol, and can therefore be considered suitable for use as a stressor in future studies. While no positive effect of CBD was observed on either the behavioural or physiological stress responses of cats during this study, it has highlighted multiple additional aspects of CBD efficacy that should be explored. Future studies that aim to investigate the use of CBD in any pet species would be advised to further consider the relationship between temperament and behavioural response to CBD, in addition to the pharmacokinetic impact of including other non-psychoactive phytocannabinoids within the CBD compound.

## Conclusion

The results from this study suggest that the use of CBD at a 4 mg/kg BW dose was not effective in positively impacting the stress response of cats who experienced travel in a cat carrier and meeting a novel person in an unfamiliar room. These events were however successful in eliciting a significant stress response in this population of cats, as evidenced by elevated plasma cortisol. Further research is required to determine if differences in cat personality traits and coping strategies masked the influence of CBD throughout this trial. Additionally, the rate of CBD absorption, and effects of both dose size and frequency on stress response should be explored—ideally across a variety of other potential feline stress-inducing situations.

## Data Availability

The raw data supporting the conclusions of this article will be made available upon reasonable request, subject to applicable legal and practical constraints.
