# Supplementary material for: Investigating the effect a single dose of cannabidiol has on measures of stress in cats when being transported in a carrier and meeting a novel person in an unfamiliar environment
Source: Front Vet Sci. 2024 Nov 4;11:1476296. doi: 10.3389/fvets.2024.1476296 (PMC11571322; doi:10.3389/fvets.2024.1476296)
Supplement: Supplementary file 1 [file Table_1.docx]

Supplementary Material 1 - Ethogram from Cat Stress Score (CSS) developed by Kessler & Turner, 1997 [34].

| **Score** | **Activity Level** | **Body** | **Belly** | **Legs** | **Tail** | **Head** | **Eyes** | **Pupils** | **Ears** | **Whiskers** | **Vocalisation** | **Activity** |
| --- | --- | --- | --- | --- | --- | --- | --- | --- | --- | --- | --- | --- |
| 1. Fully Relaxed | Inactive | Laid out on side or back | Exposed, slow ventilation | Fully extended | Extended or loosely wrapped | Laid on the surface with chin upwards or on the surface | Closed or half open, may blinking slowly | Normal | Half back (normal) | Lateral (normal) | None | Sleeping or resting |
|  | Active | Not applicable |  | No applicable | Not applicable |  |  |  |  |  |  |  |
| 2. Weakly Relaxed | Inactive | Laid ventrally or half on side or siting | Exposed or not exposed, slow or normal ventilation | Bent, hind legs may be laid out | Extended or loosely wrapped | Laid on the surface or over the body, some movement | Closed, half open or normal opened | Normal | Half back (normal) or erected to front | Lateral (normal) or forward (normal) | None | Sleeping, resting, alert or active, may be playing |
|  | Active | Standing or moving, back horizontal |  | When standing extended | Tail up or loosely downwards |  |  |  |  |  |  |  |
| 3. Weakly Tense | Inactive | Laid ventrally or siting | Not exposed, normal ventilation | Bent | On the body or curved backwards, may be twitching | Over the body, some movement | Normal opened | Normal | Half back (normal) or erected to front or back and forward on head | Lateral (normal) or forward | Meow or quiet | Resting, awake or actively exploring |
|  | Active | Standing or moving, back horizontal |  | When standing extended | Up or tense downwards, may be twitching |  |  |  |  |  |  |  |
| 4. Very Tense | Inactive | Laid ventral, rolled or sitting | Not exposed, normal ventilation | Bent | Close to the body | Over the body or pressed to body, little or no movement | Widely open or pressed together | Normal or partially dilated | Erected to front or back, or back and forward on head | Lateral (normal) or forward | Meow, plaintive meow or quiet | Cramped sleeping, resting or alert, may be actively exploring, trying to escape |
|  | Active | Standing or moving, body behind lower than in front |  | When standing hind leg bent, in front extended | Tense downwards or curled forward, may be twitching |  |  |  |  |  |  |  |
| 5. Fearful, Stiff | Inactive | Laid ventrally or sitting | Not exposed, normal or fast ventilation | Bent | Close to the body | On the plane of the body. Less or no movement | Widely opened | Dilated | Partially flattened | Lateral (normal), forward or back | Plaintive meow, yowling, growling or quiet | Alert, may be actively trying to escape |
|  | Active | Standing or moving, body behind lower than in front |  | Bent near to surface | Curled forward close to the body |  |  |  |  |  |  |  |
| 6. Very Fearful | Inactive | Laid ventrally or crouched directly on tip of all paws, may be shaking | Not exposed, fast ventilation | Bent | Close to the body | Near to surface, motionless | Fully opened | Fully dilated | Fully flattened | Back | Plaintive meow, yowling, growling, or quiet | Motionless alert or actively prowling |
|  | Active | Whole body near to ground, crawling, may be shaking |  | Bent near to surface | Curled forward close to the body |  |  |  |  |  |  |  |
| 7. Terrorized | Inactive | Crouched directly on top of all fours, shaking | Not exposed, fast ventilation | Bent | Close to the body | Lowe than the body, motionless | Fully opened | Fully dilated | Fully flattened back on head | Back | Plaintive meow, yowling growling or quiet | Motionless alert |
|  | Active | Not applicable |  | Not applicable | No applicable |  |  |  |  |  |  |  |

Supplementary Material 2 - Mean Estimate (ME) Mean Cat Stress Score, Qualitative Behaviour analysis principal components 1 and 2 (QBA PCA 1 and 2) and additional coded behaviours for the CBD and placebo treatment. Corresponding superscript letters indicate significant differences between treatments and sampling times. Upper and Lower Confidence intervals (CI) are also presented.

|  | **Treatment** | | | | | | |
| --- | --- | --- | --- | --- | --- | --- | --- |
|  | **CBD** | | | **Placebo** | | | |
|  | **ME** | **Upper CI** | **Lower CI** | | **ME** | **Upper CI** | **Lower CI** |
| **Mean Cat Stress Score** | 3.02 | 3.23 | 2.81 | | 2.99 | 3.20 | 2.77 |
| **QBA PCA 1** | -0.28 | 0.99 | -1.55 | | 0.19 | 1.47 | -1.09 |
| **QBA PCA 2** | 0.04 | 0.54 | -0.47 | | 0.08 | 0.60 | -0.43 |
| **Duration of Vocalisations (s)** | 3.93 | 8.63 | 1.79 | | 4.16 | 9.14 | 1.89 |
| **Time spent in Carrier (s)** | 151.0 | 195.2 | 106.7 | | 131.6 | 175.9 | 87.33 |
| **Time spent in Contact with Novel Person while not Receiving Encouragement (s)** | 1.25 | 2.14 | 0.74 | | 1.84 | 3.14 | 1.08 |
| **Time spent Touching the Novel Person while Receiving Encouragement (s)** | 6.90 | 10.12 | 3.68 | | 7.59 | 10.82 | 4.37 |
| **Latency to approach 1.0m (s)** | 172.9 | 227.7 | 118.1 | | 157.6 | 212.4 | 102.8 |
| **Latency to approach 0.5m (s)** | 192.5 | 247.6 | 137.4 | | 166.4 | 221.5 | 111.3 |
